# Microstructure of Rhenium Doped Ni-Cr Deposits Produced by Laser Cladding

**DOI:** 10.3390/ma14112745

**Published:** 2021-05-22

**Authors:** Paweł Kołodziejczak, Dariusz Golański, Tomasz Chmielewski, Marcin Chmielewski

**Affiliations:** 1 Institute of Manufacturing Technologies, Warsaw University of Technology, Narbutta 85, 02-524 Warsaw, Poland; pawel.kolodziejczak@pw.edu.pl (P.K.); tomasz.chmielewski@pw.edu.pl (T.C.); 2 Centre of Electronic Materials Technology, Łukasiewicz Research Network Institute of Microelectronics and Photonics, Wólczyńska 133, 01-919 Warsaw, Poland; marcin.chmielewski@imif.lukasiewicz.gov.pl

**Keywords:** laser cladding, Ni-Cr-Re clads, microstructure

## Abstract

The addition of Rhenium up to 6% to Ni-Cr alloys can dramatically improve the corrosion and oxide resistance of deposited coatings at high operating temperatures. Ni-Cr+Re layers can be successfully produced using conventional powder metallurgy, high rate solidification (HRS), or magnetron sputtering methods. However, in industrial applications, high-performance deposition methods are needed, e.g., laser cladding. Laser cladding has several advantages, e.g., metallurgical bonding, narrow heat-affected zone (HAZ), low dilution, and slight thermal damage to the substrate. In this paper, a powder Ni-Cr composite with 1% (wt.) of Rhenium was produced, then deposited onto a steel substrate (16Mo3) by laser cladding to assess the micro and macrostructural properties of the obtained layers. Besides the macro and microscopic observations, scanning electron microscopy (SEM) and energy-dispersive X-ray spectroscopy (EDS) microanalysis of the deposit and HAZ as well as microhardness measurements have been conducted. The microstructure observations revealed four subareas of HAZ gradually changing from the fusion line towards the base material. Maximum hardness occurred in the HAZ, mainly in areas closer to the clad/substrate interface, reaching up to 350–400 HV. No sudden changes in the composition of the deposit and the area of fusion line were observed.

## 1. Introduction

The surfaces of machine parts working at elevated temperatures in harsh corrosion and erosion environments are subjected to increasingly higher performance requirements, which occur in many areas of application, e.g., energy, aerospace [1], chemical, and other industries [2]. One of the widely used materials for surface layers resistant to difficult working conditions at high temperatures are Ni-Cr alloys. They have found wide application, among others, for working surfaces of turbine blades in aircraft engines [3]. Another broad area of application of these materials is also boiler surface elements used in the power industry [4], for which the protective requirements, e.g., high-temperature corrosion [5] and high-temperature oxidation [6] are particularly high.

The Ni-Cr protective layers provide excellent mechanical properties, high creep resistance, and adequate chemical resistance at elevated operating temperatures—such as high corrosion resistance. The effect of temperature on the oxidation behavior of binary Ni-Cr alloys in the CO_2_ gas atmosphere has been studied in [7] and it was shown that at 650 °C, the oxidation rates were not significantly decreased until the alloy Cr content reached 30% (wt.). At a temperature of 800 °C, the oxidation rates of Ni-5Cr, Ni-10Cr, and Ni-15Cr alloys continued to increase, while those of alloys with 20 wt.% Cr and above reduced due to protective external chromia scale formation. The oxidation resistance in the conditions of steam environments was studied in [8] for FeCrNi alloy where it was observed that the temperature increase from 800 to 1000 °C led to an increase in the oxidized surface and increased nickel diffusion. To further increase the properties of layers based on Ni-Cr alloys, doping with various compounds and elements can be used. In [9], coatings of a NiCr matrix reinforced with hard WC particles of different shapes and sizes were used for maximum thickness. It was noted that the WC content should be kept below 5–10% to avoid particle clustering. TiC particles were used in NiCrBSi composite when laser cladding of Ti-6Al-4V alloy [10]. With TiC volume fraction above 50%, clustering of TiC particles was observed. Increased laser energy resulted in a microhardness increase in the Ti+NiCrBSi clad zone. A low rhenium content was also used in addition to the clad material by replacing tungsten in nickel-based superalloys [11].

Here, the limited amount of Re below 6% (wt.) in single superalloy crystals can be utilized to aid in strengthening these alloys by solid solution means and by retarding the y’ coarsening process, which should increase the rupture live at elevated temperatures. Studies of Ni-based alloy powders with different contents of cobalt deposited on a 42CrMo steel surface using a fiber laser were described in [12]. The wear resistance increased with the increase of Co content (about 3.6 times for NiCo30 coating compared to that of the NiCo00 coating). Moreover, the addition of Al_2_O_3_ inclusions into Ni–Cr alloy matrix was used to increase the mechanical and tribological properties of the samples prepared by powder metallurgy at various alumina mass percentages from 20 to 60 [13]. The NiCr–40 (wt.%) Al_2_O_3_ composite exhibited satisfactory wear resistance over the entire temperature range from room up to 1000 °C. Depending on the scope of application and the expected properties of the material of the layer, various types of reinforcements of Ni-Cr alloys with other elements are found. For example, Ni-Cr composites with the addition of Al_2_O_3_ are often used to improve abrasion resistance [14].

In recent years, the introduction of low content (2–6%) of Rhenium in applications requiring high resistance to oxidation and corrosion at elevated temperature has been observed in Ni-Cr alloys [15]. Even a small addition of Rhenium to the Ni-Cr alloy can dramatically improve properties such as creep resistance [16], and it should be emphasized that the mechanism of material strengthening by Rhenium has not yet been fully understood and explained [17]. However, if the Re content is excessive, it tends to promote phase instabilities that lead to the formation of deleterious TCP phases during exposure to a high-temperature environment. Therefore, during the superalloy design process, the Re concentration must be controlled to stabilize the microstructure. Some research results are known where Ni-Cr superalloys (Mar-M247) without and with addition of 3 and 4.5% (wt.) Re were tested [18]. The addition of 3% (wt.) Re caused strip-like MC carbides within the grain to degenerate into discontinuous M_23_C_6_ carbides and initiated the formation of a deleterious topological closed-packed (TCP) phase within the grain interior. The addition of 4.5% (wt.) Re promoted phase instabilities led to the precipitation of large amounts of needle-like P phase in the grain interior, attributable to Re and W segregation. The 3% (wt.) Re was a critical addition to maintain optimal microstructure and phase stability and improved the ultimate tensile strength and the yield strength at room temperature and at elevated temperatures.

For research purposes, the volumetric production of Ni-Cr+Re composites may be conducted, for example, using powder metallurgy (high-pressure sintering) [17] or high-rate solidification (HRS) methods [19]. Moreover, methods based on the deposition of thin Cr-Ni-Re layers by means of magnetron sputtering are also applied [20].

From the application point of view, to produce Ni-Cr+Re layers on the surfaces of machine parts in the operating environment, it is required to use other processes to create a protective layer in a relatively short time over a larger area of the substrate. For this purpose, other methods of surface layer deposition can be applied that use different types of heat sources.

Thermal spraying methods are often used due to the high efficiency of covering large surfaces of the substrate material. High-velocity oxyfuel thermal spraying (HVOF) was used to spraying Cr_3_C_2_-NiCr composite coatings onto the 316L steel substrate to study the coating, obtaining an average value of adhesion at the level of 63 MPa [21]. The results of the investigation of NiCrRe coatings deposited by plasma spray process at the atmospheric pressure were shown in [22]. The microstructure observations confirmed the relatively low porosity of plasma-sprayed NiCrRe coatings. Both the hardness and wear resistance of the created layers were much higher than those of the base material (16Mo3). Composite materials based on Inconel 625 reinforced by WC and Cr_3_C_2_ particles have been produced via a HPDDL diode laser with a rectangular focus of the beam [23]. The heat input of the cladding of ex-situ MMC composite layers directly affects the microstructure and mechanical properties of the matrix material and affects the wear resistance of the coating. The results have shown that the clads reinforced with irregular WC particles showed a significantly higher erosive resistance than the coatings reinforced by spherical WC particles.

Recently, other methods utilizing a laser beam have also been introduced, such as laser surface alloying. During the interaction of a laser beam with a workpiece, an alloying material is introduced simultaneously into the beam area. In this process, the modified material is entirely made, e.g., Ni-Cr alloy (Inconel), and doped with a material with very precisely selected process parameters to obtain a layer with specific properties. In the study [24], Inconel 718 alloy was doped with Rhenium by laser surface alloying (LSA). Two coating compositions were studied with 14 and 28% (wt.) of Rhenium, and it was found that the layer alloyed with 28 wt.% Re has 160% higher hardness, 82% lower sliding wear rate, and 25% higher abrasive wear resistance index compared to 18% Re. The laser ablation process can also be used for thin film production, as shown in [25] for hydroxyapatite (Ca_10_(PO_4_)_6_(OH)_2_), which is biocompatible ceramics. An Nd; YAG laser operating at 355 nm produced coatings 3–6 μm thick that adhere strongly to the Ti6Al4V substrates.

Other laser-assisted coating techniques of producing clads can include laser surface cladding (LSC), laser-assisted plasma spraying (LPS), laser chemical vapor deposition (LCVD), and other techniques, e.g., laser engineered net shaping (LENS) [26].

Both continuous and pulsed cladding may be utilized. In [27] a CoNiTi medium-entropy alloy coating was produced onto pure Ti substrate by the pulsed laser cladding. The coating with good metallurgical bonding was characterized by five times higher hardness than the substrate, which is attributed to second-phase hardening from Ti_2_Ni and Ti_2_Co intermetallic compounds.

According to the general behavior of abrasive wear, the wear resistance of metallic materials is often proportional to the Vickers hardness, which allows us to expect an enhanced wear resistance for the laser cladded coatings. The Ni45 + high-carbon ferrochrome composite coatings with carbon ferrochrome ranging from 10 to 50 wt.% were produced by laser cladding on an ASTM 1045 steel substrate [28]. The highest hardness of the clad was achieved with a 30 wt.% high-carbon ferrochrome content, and the microhardness of the composite coating was 2.4 times higher than that of the 1045 substrate. Moreover, the best wear resistance was obtained when the high-carbon ferrochrome content was 30 wt.%.

Composite layers based on nickel alloys are often used in laser cladding processes. In [29], a Ni–20Cr coating was deposited on a molybdenum substrate by a continuous wave CO_2_ laser cladding. It was found that the oxidation behavior of the laser clad Ni–20Cr coating can effectively prevent oxidation of molybdenum at 600 °C in the air. The defect-free Ni–20Cr coatings can be obtained by properly selecting the processing parameters. Another factor influencing the use of laser cladding of nickel alloys is an attempt to replace stellite layers with them, especially in a nuclear industry, where cobalt and boron cannot be used. In work [30] (Ni-5Al)-5Mo, (Ni-5Al)-15Mo and Ni-15Cr-32Mo have been cladded onto 0.15% C-steel by blown powder laser cladding process together with a Stellite 6 alloy for comparison. A slight increase in hardness was recorded for all Nickel-based composite coatings, but much higher wear resistance was achieved for the Ni-15Cr-32M than Stellite 6. That means the Co-B free Ni-Cr-Mo alloy can be a favorable alternative of stellite for laser cladded layers. The laser cladding process enables efficient production of surface layers with a low dilution, ensuring the preservation of the layer properties corresponding to the materials applied. It does not require the use of expensive substrate materials, and in addition, the produced layers obtain a good metallurgical bonding to the substrate [31].

This paper presents research on the microstructure of composite layers made of Ni-Cr alloy doped with a small amount of Rhenium produced using a laser beam in the laser cladding process. The addition of Rhenium should substantially improve the corrosion and oxidation resistance of the clad at high operating temperatures. The laser cladding was used as a high-performance deposition method which is required in many industrial applications. Due to high material costs, repairing parts of gas turbines, boilers, pumps, metal forming tools, etc., with Ni-based superalloys becomes desirable. The study of laser cladding of Ni-Cr+Re composite has not been previously reported so far. Therefore, the conducted research should bring new data on the structural properties of Ni-Cr-Re layers produced by laser processing.

In the applied approach, the initially prepared Ni-20Cr powder alloy with 1% (by weight) rhenium content was fed into the laser beam stream interacting with the 16Mo3 steel substrate. As a result of remelting and mixing powder components with the substrate material, clads with a specific dilution were obtained, then subjected to microstructure investigations to assess the effects of remelting and the structure of the obtained layers.

## 2. Materials and Methods

**Figure 1:**
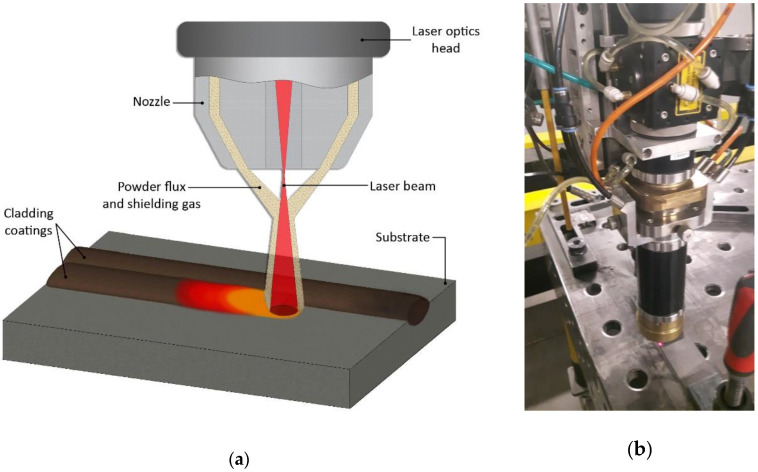
Cross-section diagram of the powder feeding head (**a**), picture of Laserline COAX head (**b**).

For the cladding of Ni-Cr+Re, a 4 kW diode laser Laserline LDF 4000-30 (Laserline GmbH, Mülheim-Kärlich, Germany) with a Laserline COAX (Figure 1) powder cladding head with the following parameters was used: focus 13 mm, fiber optic 1000 µm, stand-off distance 15 mm, the round spot with a diameter of 4.2 mm. A shielding gas in the form of argon with a constant flow rate 7 L/min. was used.

The coatings were generated on flat substrates of 16Mo3 steel (EN 10216-2) with dimensions of 100 mm × 50 mm × 4 mm. It is chromium-molybdenum boiler steel, adapted to work at elevated temperatures, used in the construction of key elements of power boilers (including steam superheaters). The steel hardness is 155 ÷ 160 HV_10_, the structure is ferritic-pearlitic, with ferrite grains of 8 ÷ 12 μm. The corresponding chemical composition of this steel is detailed in Table 1.

Commercial Ni-20Cr (Amdry 4535) spheroidal, gas atomized powder, from Oerlikon Metco Europe GmbH Sp. z.o.o., Poznań, Poland), with a particle size above 45 µm was used as a precursor material for the coatings with chemical composition (Data Sheets DSMTS-0109.2) presented in Table 2.

**Figure 2:**
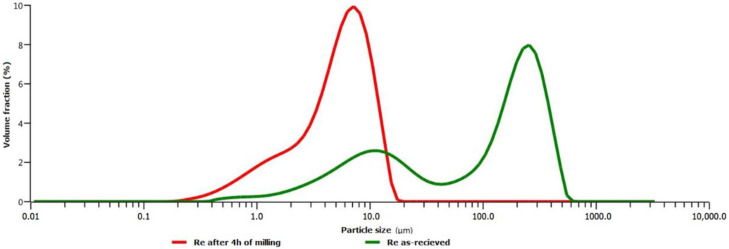
Comparison of the particle size distribution of Rhenium powder in as-received state and after 4 h of milling.

The rhenium powder 99.7% purity (KGHM ECOREN S.A., Legnica, Poland) with an average particle size of 138 µm was used. The particle size distribution was obtained using a laser diffraction technique (Malvern Mastersizer 3000 analyzer, Malvern Panalytical Ltd., Malvern, UK) that allows measurement of particle size distributions from 0.01 μm up to 3.5 mm and visualizing the results with dedicated software (Figure 2). The powder was then milled for 4 h, after which the average particle size was reduced to 5.41 μm and used as such as reinforcement for the NiCr-based composite.

**Figure 3:**
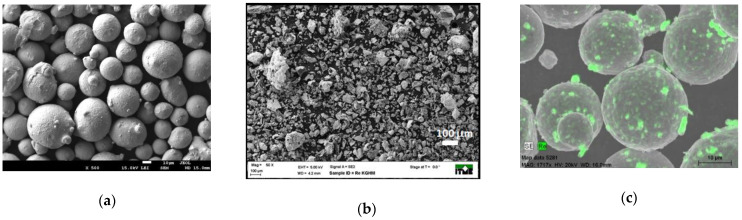
SEM micrograph showing particle size and morphology of (**a**) base powder Ni-20Cr, (**b**) Rhenium powder particles and (**c**) composite Ni-20Cr+Re particles (green color) after 4h of ball mills employed in this work.

The powder used for the final cladding in this investigation was made by mixing in WC chamber with balls (Fritsch Pulverisette 6 planetary mill—FRITSCH GmbH, Idar-Oberstein, Germany) base powder Ni-20Cr (Figure 3a) with 1% (wt.) of Rhenium (Figure 3b). The powder preparation parameters were as follows: rotation 100 rpm, ball-to-powder weight ratio (BPR) 5:1, duration 4 h, balls diameter 10 mm, amount per chamber 100 g. Figure 3c presents the composite Ni-20Cr+Re produced after 4 h of milling.

The substrate surfaces were blasted, and the surface contaminants were cleaned with Acetone prior to laser processing. The roughness parameters were adjusted to the grain size of the clad material powder in order to ensure the highest possible adhesion of the applied coating.

The first series of tests using the Ni-20Cr alloy was used to determine the range of process parameters ensuring the correct course of the process and the formation of the clad. Five sets of samples with Ni-20Cr+Re layers were produced for increasing laser power from 1500 to 2700 W with a step of 300 W, cladding velocity between 15 and 25 mm/s, and powder feed rate between 2.5 and 5 rpm at a constant argon flow rate of 7 L/min. All cladded samples were tested by micro and macroscopic observations to narrow the cladding parameters for the final samples chosen for microstructure investigations.

**Figure 4:**
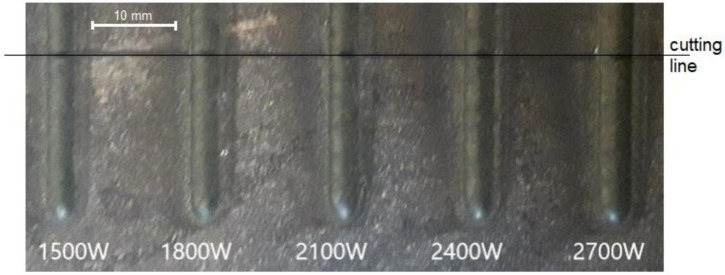
Macroscopic morphology of coatings prepared by ring beam of single bead coatings at a feeding rate of 5 rpm, laser power was changed from 1500 W up to 2700 W.

The final clads were produced as a single bead for macro and microstructure characterization purposes. The main parameters of laser cladding were laser power and powder feeding rate. The laser power was varying between 1500 W and 2700 W in five steps to study the effect of bead geometry. The view of single beads cladded at different laser power is shown in Figure 4. The feeding rate was kept constant at 5 rpm, which corresponds to about 25 g/min. for all produced clads, and the cladding speed was set to 15 mm/s. We may observe that with the increase of laser power, the surface of the coating gradually becomes smooth, while the width of the coating increases.

The microscopic analysis of produced clads was conducted on transverse cross-sections of the single bead coating using Olympus SZX9 (Olympus Corporation, Tokyo, Japan) optical microscopy. The section planes were polished and etched with 5% Nital solution for 10 s.

Scanning electron microscopy (SEM) JEOL JSM-7600F (Jeol Ltd., Tokyo, Japan) equipped with energy dispersive X-ray spectroscopy (EDS) X-MaxN detector type SDD (Oxford Instrument, Abingdon, UK) and X-ray diffraction (XRD) Philips X’Pert diffractometer using CuKα (Philips, Almelo, The Netherlands) were used for the microstructure characterization and phase analysis of coatings. A microhardness test along the cross-section (start from the coating across HAZ towards the parent material) was performed using a Vickers Leitz Wetzlar 8375 (Ernst Leitz GmbH, Wetzlar, Germany) tester with the 200 g load.

## 3. Results

### 3.1. Macro Morphology

When laser cladding metals, the depth, chemical composition, microstructure, and associated properties of the alloyed zone depend on the suitable choice of laser type and processing parameters, i.e., incident power or energy, beam diameter, shape or profile, interaction time, thickness, chemical composition and physical properties like absorption coefficient, thermal conductivity, melting point and density of materials used [32].

The microstructure of solidifying clad metal at the moment is related to the cooling rate in such a way that the higher the cooling rate, the finer the microstructure of the clad. The cooling rate (i.e., the temperature gradient) decreases from a maximum value at the clad symmetry vertical axis to zero at the edge of the molten pool. Moreover, it decreases from a maximum value on the clad surface to a minimum value at the bottom of the molten pool. Such variation in the cooling rate causes changes of the solidified microstructure of the clad metal.

**Figure 5:**
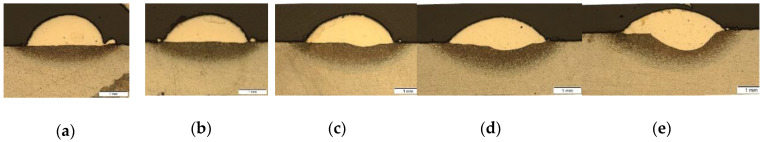
Cross-sections of a single Ni-20Cr+Re bead produced by laser cladding on 16Mo3 steel substrate at different laser power values: (**a**) 1500 W, (**b**) 1800 W, (**c**) 2100 W, (**d**) 2400 W, (**e**) 2700 W.

To find the correct shape and thus adequate quality of laser clads, it was important to study Ni-20Cr+Re bead geometry. The macroscopic morphology of the laser cladded single beads was observed by optical microscopy. Samples with the clads were cut at a distance of approximately 18 mm from the start of the bead (Figure 4). The Ni-20Cr+Re beads were produced by the laser power from 1500 up to 2700 W, as shown in Figure 5.

Analyzing the geometrical shape of produced Ni-20Cr+Re beads, we may suspect that there is a direct correlation between the laser irradiance and the clad dilution, which is twice as high in the bead made at 2400 W laser power compared to the bead obtained at 2100 W laser power. For low values of laser power used (1500 and 1800 W), the samples revealed almost no dilution zones. As shown in Figure 5, there are no cracks and porosities visible in the clads and at the interface between the clad and the substrate.

**Figure 6:**
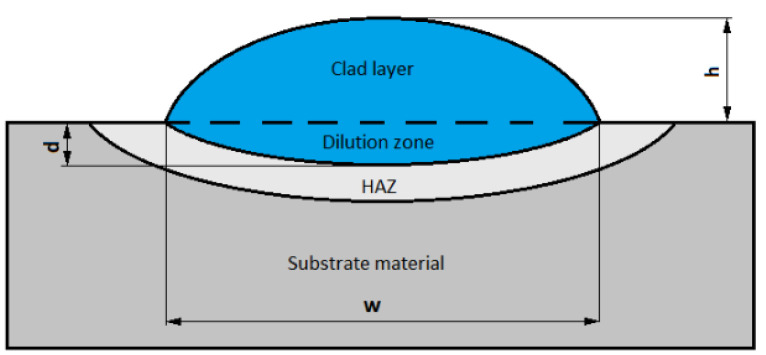
Diagram showing geometrical parameters of a laser cladded single bead: height (h), depth (d), width (w).

To measure the geometrical parameters of the clad i.e., height, depth, and width (Figure 6), the samples produced with a single Ni-20Cr+Re bead were measured, and the amount of dilution (D) was calculated using the following formula:

(1) $$D=\frac{\mathrm{dilution}\;\mathrm{zone}\;\mathrm{area}}{\mathrm{dilution}\;\mathrm{zone}\;\mathrm{area}+\mathrm{clad}\;\mathrm{layer}\;\mathrm{area}}\times 100\%$$

The results of measurements and calculated dilution are shown in Table 3. The lowest laser power used (1500 and 1800 W) revealed no dilution. The further increase of laser power created better penetration of the clad into the metal substrate and increased the height and width of the clad geometry. The highest dilution reached 20.8% at the highest laser power, but preferred dilution values should be kept as low as possible to obtain optimal properties in the cladded deposit. Therefore, preferable dilution and clad geometrical parameters seem to be obtained at the laser power of 2400 W.

### 3.2. Microstructure Observation

**Figure 7:**
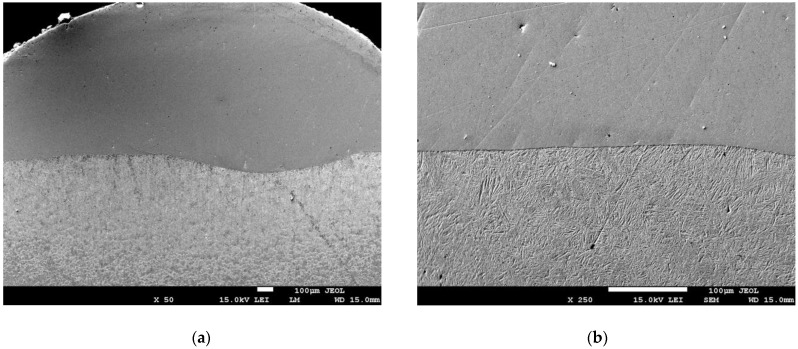
SEM images of Ni-20Cr+Re deposit (**a**) and interface (**b**) obtained by laser cladding at the power of 2400 W.

In most cases, the microscopic analysis revealed no changes in the produced Ni-20Cr+Re composite clads (Figure 7). The interface between the clad and substrate is clearly visible. We may see no cracks or pores inside the deposited material. Only the heat-affected zone (HAZ) is characterized by a dissimilar structure changing towards the base material.

**Figure 8:**
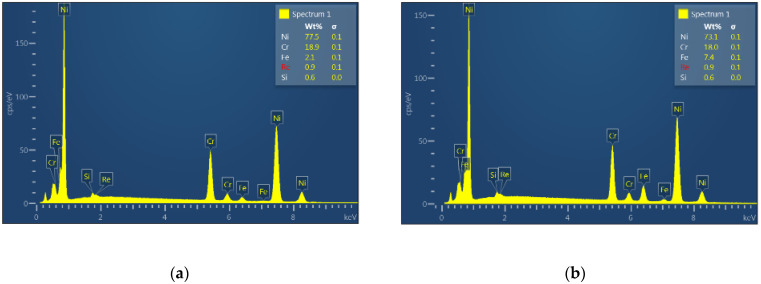

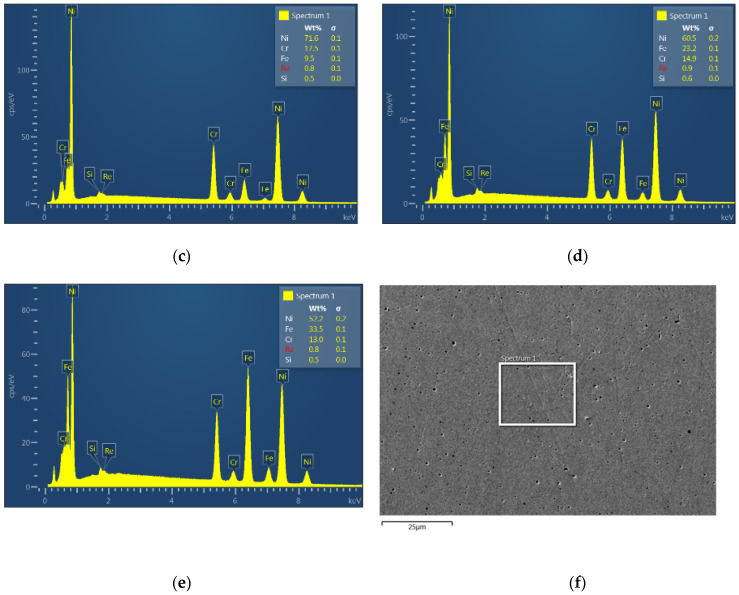
EDS spectrum obtained from the Ni-20Cr+Re clad produced at different laser power: (**a**) 1500 W, (**b**) 1800 W, (**c**) 2100 W, (**d**) 2400 W, (**e**) 2700 W, (**f**) clad area taken for the analysis.

Figure 8 shows the analysis of the distribution of elements in the area of the clad (marked with a rectangle in Figure 8f) for samples obtained at all laser power values from 1500 to 2700 W. The position of the EDS measurement was set at a distance of around 120 μm from the interface at the clad side. In all samples, the analysis shows the content of Rhenium in the clad in the range of 0.8–0.9%. The proportion of nickel and chromium gradually decreases with increasing laser power from 77.5% to 52.2% at the highest power. This decrease is accompanied by an increase in the proportion of iron from the steel substrate to 33.5% for the highest power. From the point of view of the service properties of the tested layers, the proportion of iron in the clad should be as low as possible while maintaining the appropriate dilution and clad shape. For this reason, the clad obtained for a laser power of 2400 W was selected for further microstructure investigation.

**Figure 9:**
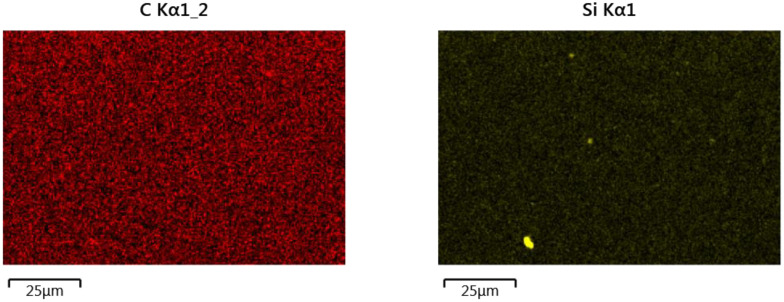

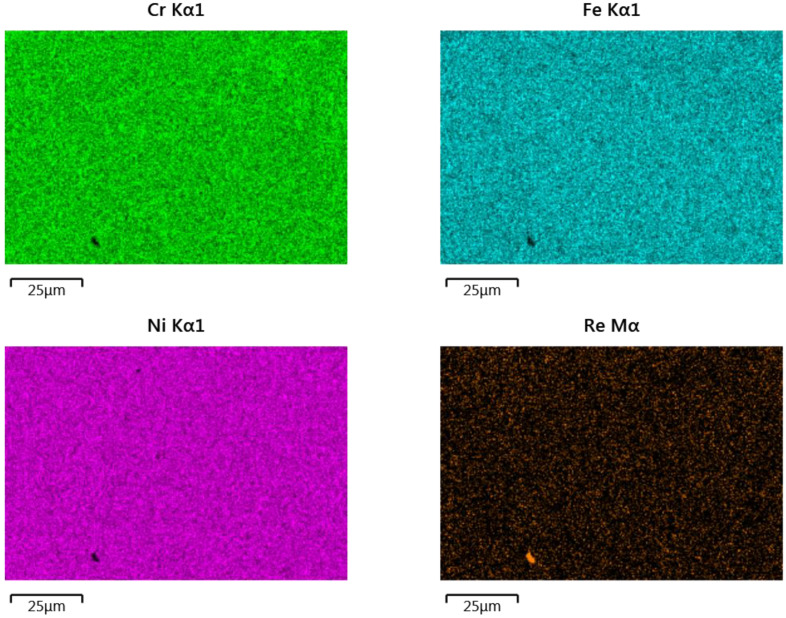
Surface distribution of elements in the laser cladded Ni-20Cr+Re bead at 2400 W laser power.

The results of the analysis of the element surface distribution in the clad material taken from the sample produced at 2400 W laser power is shown in Figure 9. The presented images show a fairly even distribution of elements in the area of the composite Ni-20Cr+Re deposit. The dominant parts here are nickel, chromium and iron. The rhenium share in the clad layer is insignificant (slightly below 1%) and its distribution in the analyzed area can also be described as evenly distributed.

**Figure 10:**
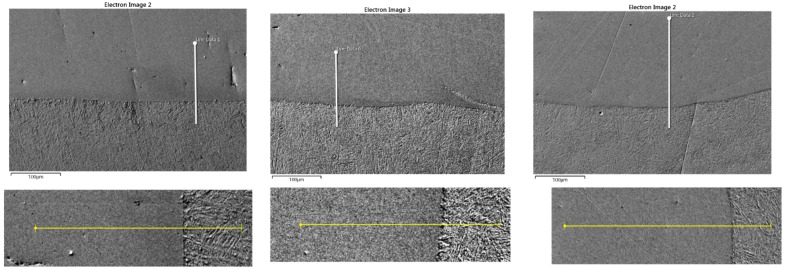

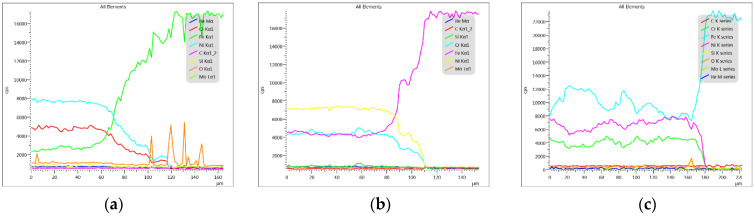
Linear element distributions in the cross section perpendicular to the fusion line of the laser power of (**a**) 2100, (**b**) 2400, (**c**) 2700 W.

**Figure 11:**
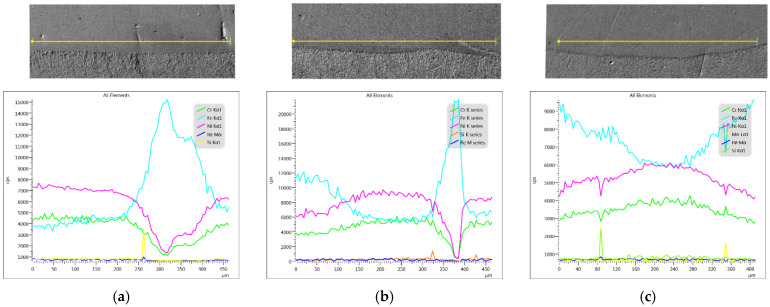
Linear element distributions in the cross-section parallel to the fusion line for laser power of (**a**) 2100, (**b**) 2400, (**c**) 2700 W.

The next two figures show the linear distributions of the elements in a cross-section perpendicular to the fusion line (Figure 10) and in a cross-section parallel to the fusion line on the side of the clad composite (Figure 11). Linear element distributions are presented for the clads obtained at the highest laser powers (2100, 2400, and 2700 W), for which the dilution value was greater than zero.

In the case of the distributions perpendicular to the fusion line, the content of nickel and chromium in the area at the fusion line is decreasing. At the same time, the iron content increases already at a distance of approximately 20–30 μm from the fusion line for all laser powers. The level of iron in the clad systematically increases with the increase in laser power (it doubles after each increase of the laser power). More significant fluctuations in the distribution of iron in the clad are clearly visible in a sample obtained at the power of 2400 W. The rhenium level is kept more or less constant low for all laser powers.

The distribution of elements in the cross-section parallel to the fusion line shows some variability in the content of iron, nickel and chromium (Figure 11). The increase in iron content is accompanied by a decrease in the share of nickel and chromium. For the highest laser power of 2700 W, where the curvature of the fusion line is visible in the SEM photo (Figure 11c), the increase in iron content occurs where the measuring line is closest to the fusion line, i.e., at its beginning and end. It is also visible to a lesser extent in Figure 11b.

**Figure 12:**
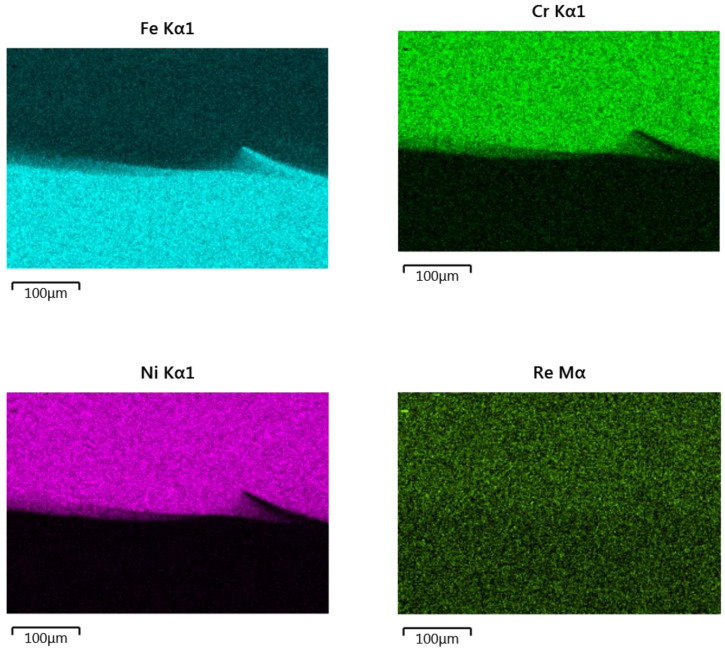
Surface element distributions at the interface in the Ni-Cr20+Re clad sample (shown in Figure 11b) produced at 2400 W laser power.

In the case of clad produced at the power of 2400 W (Figure 11b), the SEM picture clearly shows a fragment of the substrate that sharply cuts in the area of the clad, which results in a steep increase in iron content and a decrease of chromium and nickel on the measuring line crossing this area. This sudden change in the element distribution is also visible in Figure 12, showing the element surface distribution in this area. Analyzing the area close to the fusion zone, we can see a significant expansion of the fusion line, promoting excellent metallurgical bonding between the deposit and the substrate material. This in turn should ensure good properties of the obtained clad. The elements surface distribution (Figure 12) also shows a gradual change of element concentration at the fusion zone. The rhenium content for all analyzed clads remains at a more or less constant low level over the entire length of the measuring section line.

**Figure 13:**
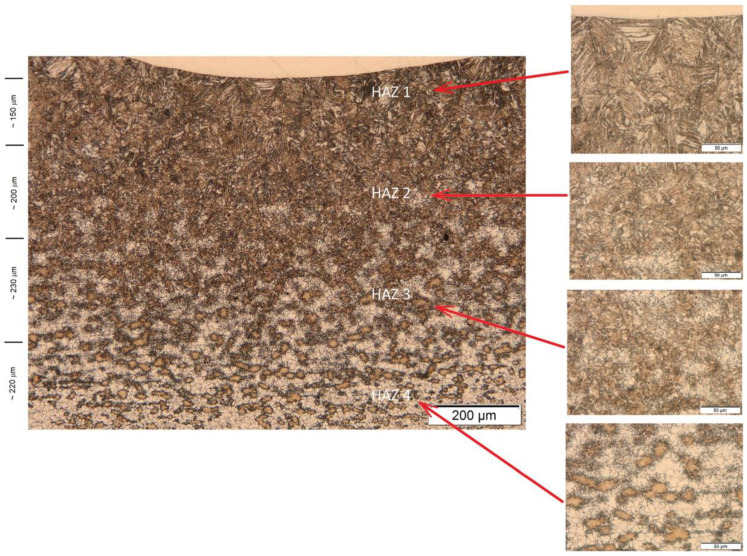
The micrograph showing the interface between the Ni-20Cr+Re coating and the 16Mo3 steel substrate obtained at the 2400 W laser power. Four different microstructure subzones (HAZ 1, HAZ 2, HAZ 3, HAZ 4) can be distinguished within the heat-affected zone.

The typical microstructure of the heat-affected zone (HAZ) located at the interface between the Ni-20Cr+Re clad and the steel substrate is presented in Figure 13. This structure represents the sample obtained using 2400 W laser power. The other samples produced with the remaining values of the laser power were characterized by similar HAZ structure but differed by the clad geometric parameters. From the dilution point of view, the clad geometry produced at 2400 W seemed to have adequate geometry and was chosen for further microstructure analysis.

The HAZ is a complex area subjected to thermal effects during a sudden and short heating period and followed by cooling in the air of each bead formed. This type of heat treatment is similar to rapid cooling in the air from different temperature ranges. Immediately, below the deposit/HAZ interface, the microstructure is transformed to an acicular ferrite and lath martensite/bainite. The coarsening near the clad/HAZ interface is observed, and further away from the clad/HAZ interface, the grain size becomes finer due to decreased temperatures.

Analyzing the HAZ, we may distinguish four distinct subzones that we called HAZ 1, HAZ 2, HAZ 3, and HAZ 4 (Figure 13). As we can see the whole structure of the HAZ changes gradually from the fusion line to the substrate material and a diverse microstructure was observed: from martensitic, through martensitic–bainitic, to ferritic–bainitic microstructure. The HAZ1 subzone with a length of up to approximately 150 μm presents a martensitic structure. The next three subzones have the length of around 200 μm, and their structure is gradually changing. We can see more and more large grain fractions with clearly visible boundaries. For a more detailed characterization of the changes in HAZ, EDS analysis was performed on the samples.

**Figure 14:**
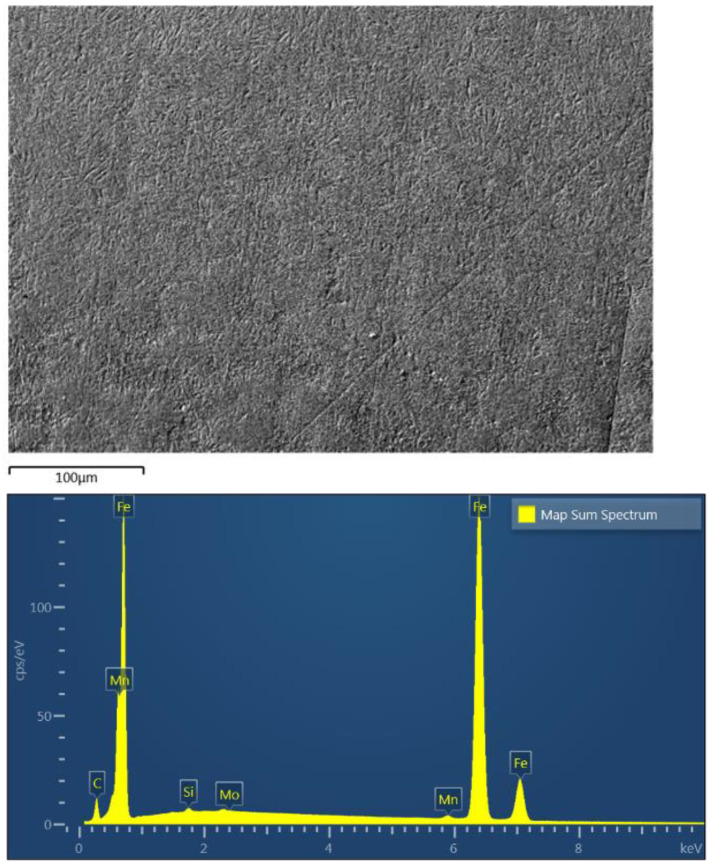
EDS spectrum registered in the HAZ of a sample cladded at 2400 W laser power at a position close to the interface (HAZ1 + HAZ2 subareas -upper photo).

The chemical composition of HAZ was studied by EDS spectrum analysis. Figure 14 presents the EDS spectrum taken from a region located in the HAZ1 subarea close to the interface and the adjacent HAZ2 subzone. Qualitatively, the registered spectrum is similar for all clads produced at different laser power. It presents iron, carbon, manganese, molybdenum, and silicon for the substrate as the main elements. The elements coming from the deposited material are not observed in this region. As previously shown, the HAZ is characterized by a gradually varying structure depending on the distance measured from the fusion line. Still, it remains unchanged in terms of chemical composition in relation to the substrate and does not depend on the laser cladding parameters used.

**Figure 15:**
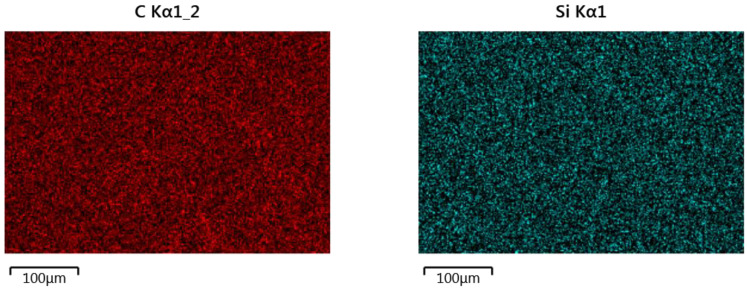

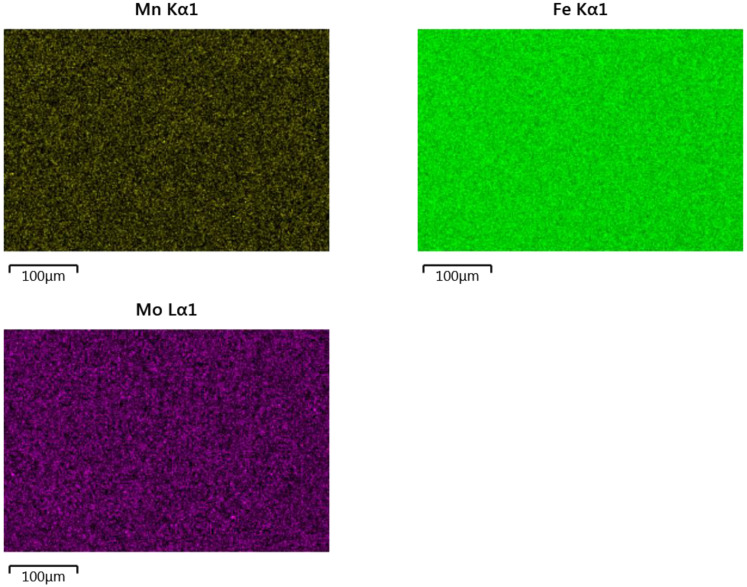
Surface element distributions in the HAZ1 subarea (shown in Figure 14) produced at 2400 W laser power.

The EDS element surface distribution presented in Figure 15 shows the uniform distribution of elements in the analyzed HAZ1 subarea. Similarly, a uniform distribution of elements was obtained for subsequent zones from HAZ2 to HAZ4.

**Figure 16:**
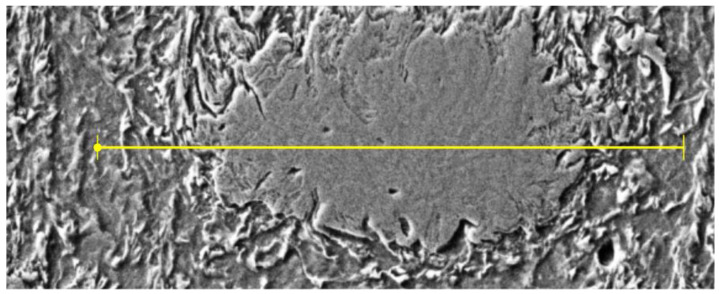

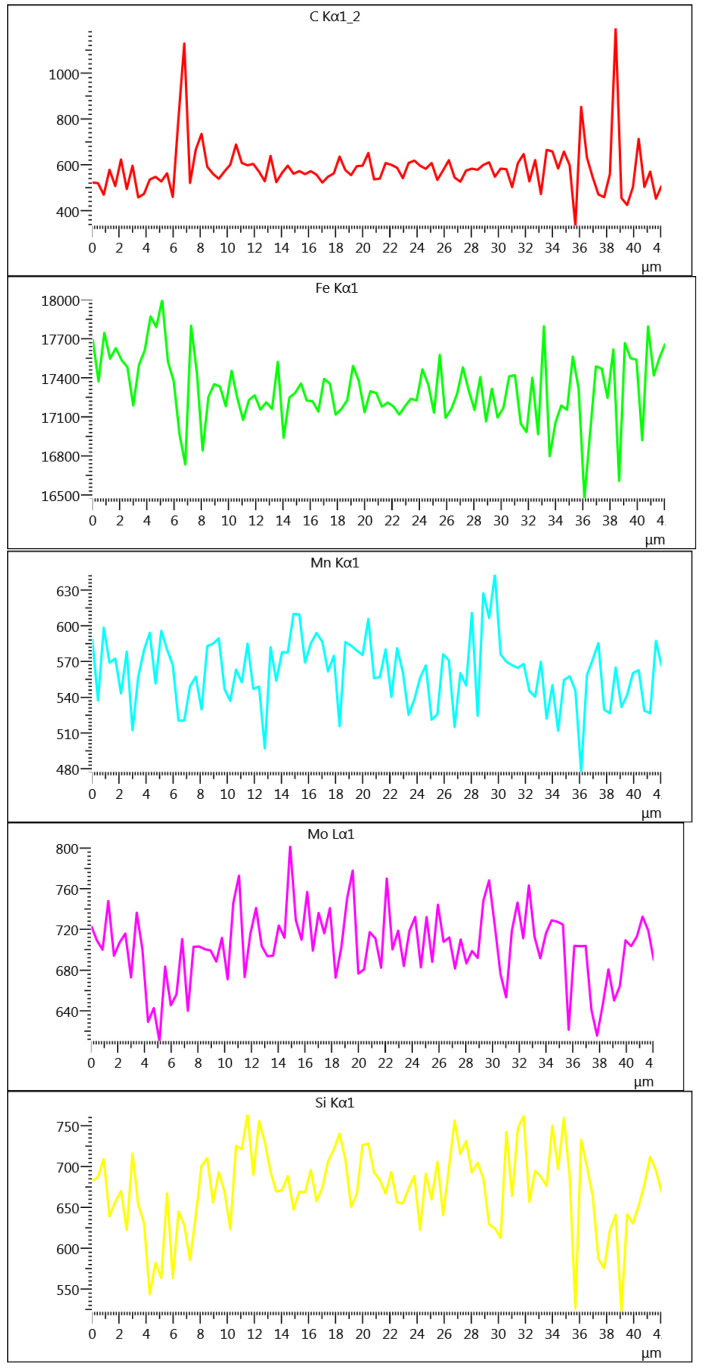
EDS analysis of dark fields from subarea IV.

After further observations of changes in the substrate’s structure in the HAZ, an analysis of the chemical composition of dark subareas found in zone HAZ 4 (Figure 16) was performed. The longest size of the presented area does not exceed 40 µm. The EDS analysis showed no significant changes in the entire analyzed area. Only a specific increase in carbon content was observed at the boundaries of the dark subarea, with a simultaneous decrease in ferrite and molybdenum contents. These results do not indicate that new phases may be formed in the area. Element EDX surface maps were also obtained for the area presented in Figure 16, which showed an almost uniform distribution of all detected elements (C, Fe, Mo, Mn, and Si). Hence, they are omitted here.

**Figure 17:**
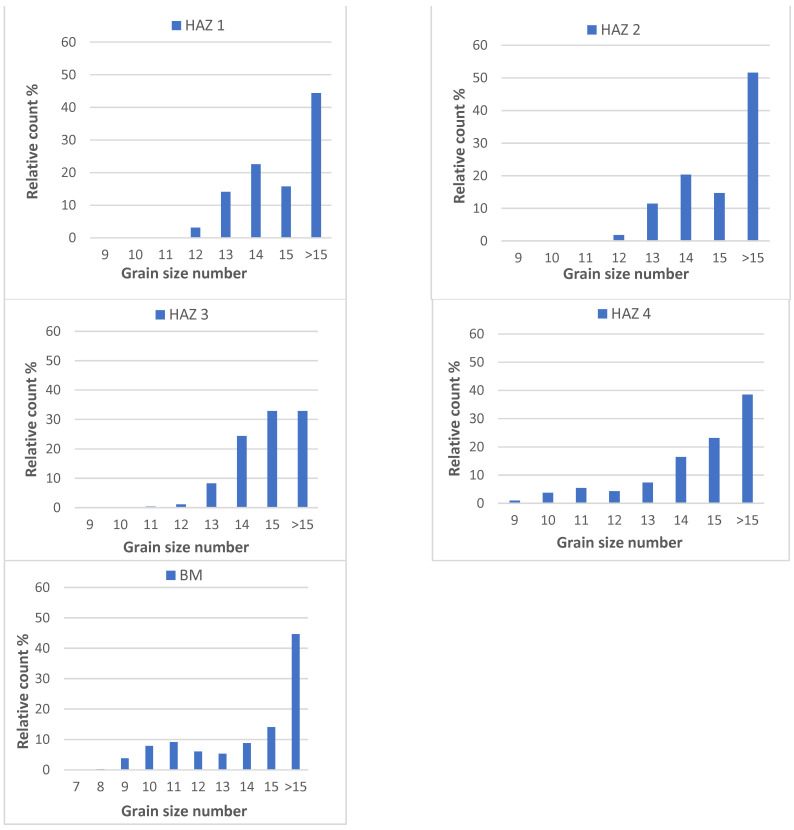
Percentage grain size distribution in the heat-affected zone divided into four subareas (HAZ 1 to HAZ 4) and in the base material (BM).

The microstructure images in the HAZ show an apparent difference in grain size in each HAZ subarea. This can lead to changes in hardness in individual zones. The grain size distribution within every four subareas in the heat-affected zone has been calculated of the rectangular area 200 × 200 μm^2^. The percentage grain size distribution shown in Figure 17 was calculated with the Olympus microscope dedicated software using the planimetric method according to the ASTM E 112–13 standard.

Based on the pictures of the microstructure in the HAZ 1 and HAZ 2 zones, an acicular structure with relatively large grains is visible. Going further towards the HAZ 3 area and then to HAZ 4, we can see the appearance of grain fractions with a larger diameter, which are visible on the histograms represented by G numbers 9, 10, and 11. In these zones, the grain boundaries are quite clearly marked, in contrast to the HAZ 1 and HAZ 2 regions, for which the results may be burdened with a more significant error due to the less distinct boundary between the grains in the structure. In the area of the base material, the grain size distribution shows the appearance of fractions with higher grain size (G number 7–11).

**Figure 18:**
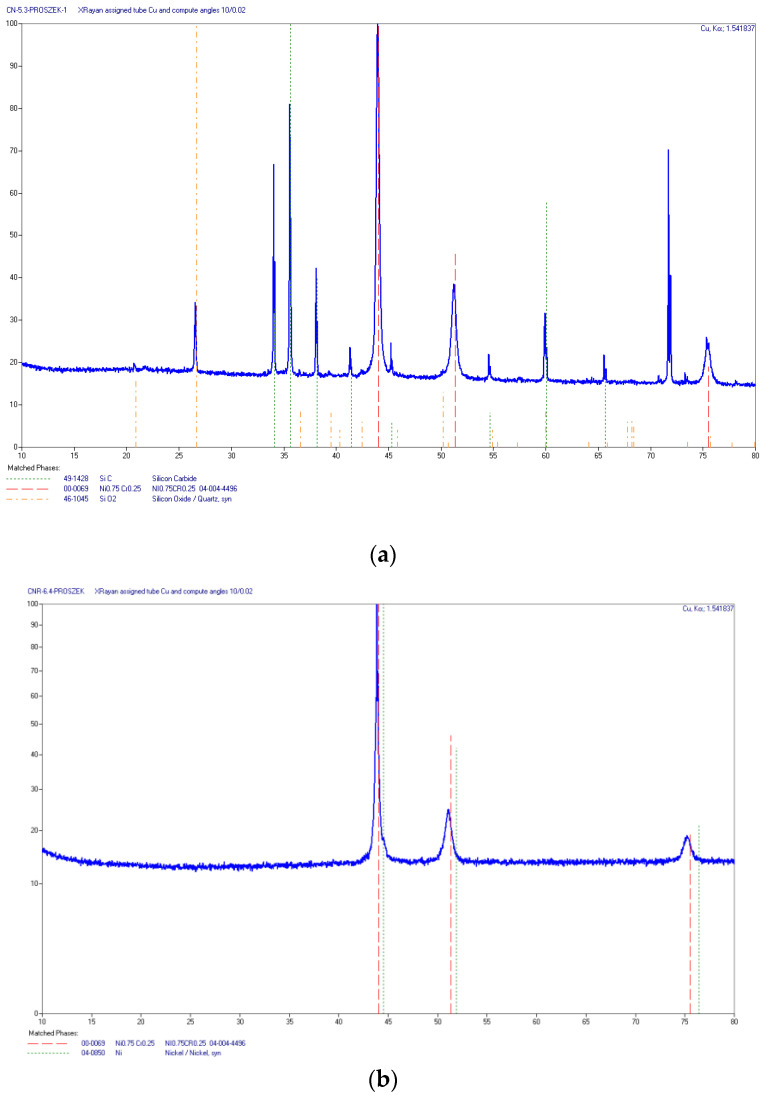
XRD patterns obtained for produced deposits: (**a**) Ni-20Cr, (**b**) Ni-20Cr+Re.

Beside EDS microanalysis, X-ray diffraction analysis was performed to compare the deposit made of Ni-20Cr with the Ni-20Cr+Re composite clad. The obtained X-ray diffraction patterns (Figure 18) looked similar for both deposit materials, mainly due to the low content of Rhenium (below 1% wt.). However, Rhenium itself was not found due to its low content in the composite. The XRD method requires at least 2% of an element in the sample to be identified. No other phases have been registered in the clad area beside SiC that probably came from the material used for sample preparation.

The provided diffraction patterns from the clad area show the phase composition of the coating which consists predominantly of a Ni-Cr alloy matrix. The linear distribution of elements along the fusion line (Figure 11) on the side of the clad does not indicate any sudden changes, which could suggest the presence of new phases. Moreover, the surface distributions of the elements seem quite homogeneous in the area of the clad (Figure 12). The lack of observed changes in the structure of the clad, which could indicate the potential occurrence of new, secondary phases, could lead to further development of research into the structure of selected areas of the clad. Still, the performed investigations using EDS and X-ray analyses did not indicate the existence of such phases in the clad and the substrate material.

### 3.3. Microhardness Analysis

The microhardness analysis was performed using Leitz Wetzlar 8375 hardness testing machine under a load of 200 g. The hardness distribution was measured along the center of the vertical axis starting from the clad surface through the fusion line, HAZ, and base material. Figure 18 shows the microhardness distribution of cladded samples for all the laser power levels applied.

**Figure 19:**
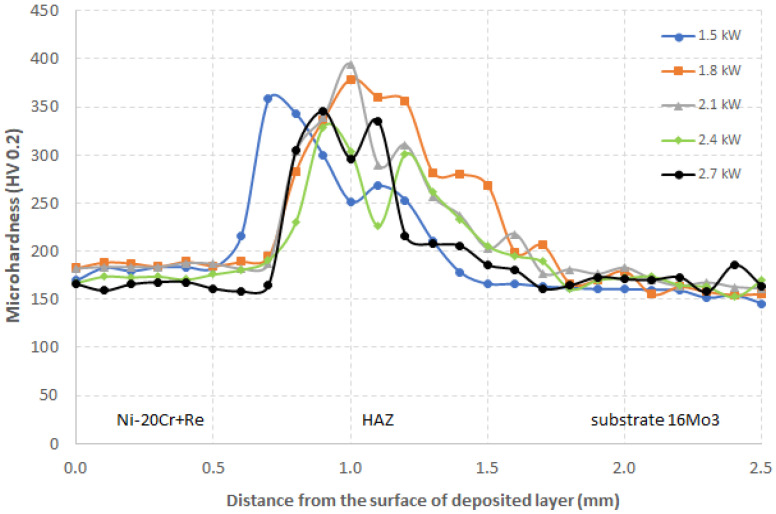
Microhardness distribution at the cross-section of the deposited Ni-20Cr+Re layer produced by laser cladding with different power level.

The increase in laser power affects only the geometry and dilution of the resulting clad, as was presented in Figure 5 and Table 3. From Figure 19, we may say that the hardness measured inside the Ni-20Cr+Re clad has a constant value of about 186 HV for laser power between 1500 and 2100 W. With a further increase in the laser power to 2400 and 2700 W, the hardness in the deposit decreases to 166 HV.

A significant increase in microhardness was observed in the HAZ, especially in HAZ 1 and HAZ 2. It reached over 350 HV for 1500 and 1700 W laser power and almost 400 HV for 2100 W power. A further increase in laser power reduced the maximum hardness in the HAZ to approximately 330–340 HV. This increase of hardness in the HAZ results from changes in the structure arising at the heat-affected zone.

Microhardness changes directly depend on the laser power used for cladding. With the increase in power, the dilution and width of the clad increase with the simultaneous rise in the area of the substrate material with structural changes caused by the thermal interaction of the laser beam. For laser power of 1500 and 1800 W, there is practically no dilution with the substrate, and the unit area of a clad is much smaller than a clad produced at 2700 W. Moreover, the relatively low laser power/density and high scanning speed corresponded to the high cooling rate and high solidification rate of the molten pool, thereby increasing the undercooling of the solidification front [33]. These factors probably change the heat input conditions at the clad/substrate interface and promote a steeper increase of hardness for lower laser power.

In the HAZ sub-area HAZ 3 and HAZ 4, hardness decreased to the base material, in which it was about 170 HV. The change in hardness from the HAZ proceeds in a relatively mild way, which is beneficial from the point of view of the properties (or gradient properties) in this region.

By analyzing the hardness distribution in the HAZ area, it can be seen that the sudden increase in hardness in the HAZ 1 zone is related to the appearance of an acicular structure characterized by a greater share of grains from the G number in the range 13–15 (Figure 17). This corresponds to an average grain diameter in the range of below 2.8 up to 4.0 μm according to the ASTM E112–12 standard. In addition, in the next HAZ 2 subarea, the grain size distribution is very similar to the distribution in HAZ 1, which can be compared in the first two histograms in Figure 17. The area of increased hardness (above 300 HV) covers a zone with greater width, up to approximately 0.5–0.6 mm for the analyzed laser powers. Only for the lowest laser power of 1.5 kW, the total width of these zones is slightly smaller, probably due to the lower amount of heat introduced into the material.

Only in the HAZ 3 zone and further in the HAZ 4 zone, larger grain size fractions (G number from 9 to 11) appear on the histograms, corresponding to the average grain size in the range of 7.9–15.9 μm. The total width of both these zones is approximately 0.5 mm. In the HAZ 3 zone, there is a regular grain structure with visible precipitates, the amount of which gradually decreases when approaching the HAZ 4 region and completely disappears in the area of the base material. The EDS analysis of these precipitates is presented in Figure 16. The hardness in these two zones ranges from 210–300 HV and systematically decreases to the hardness level of the base material (155–165 HV), which is characterized by a ferritic-pearlitic structure with ferrite grains of G number mostly between 9 and 15 (2.8 ÷ 15.9 μm).

**Figure 20:**
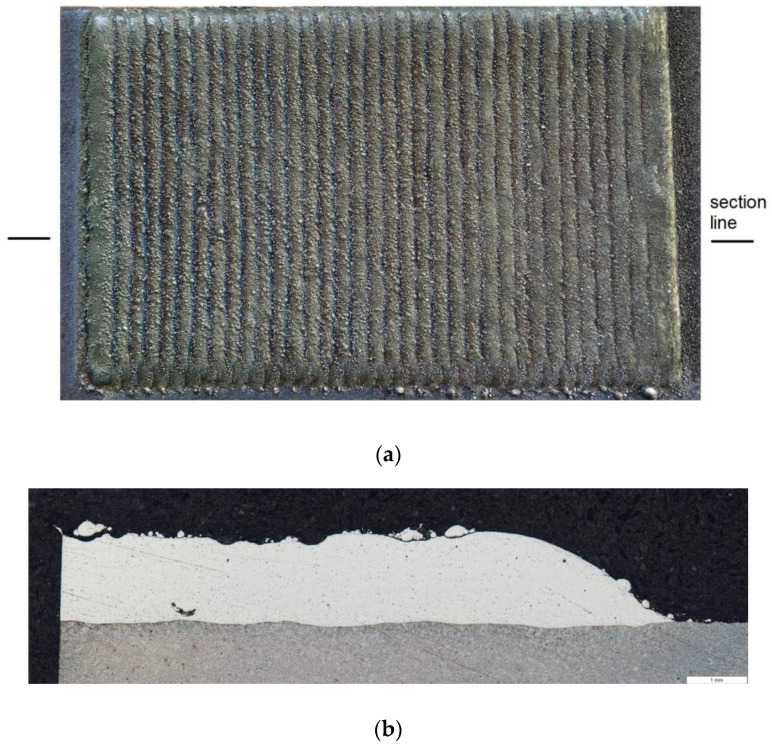
Ni-20Cr+Re multi-bead deposit sample produced at the laser power of 2400 W: (**a**) a photo of the clad surface (100 × 50 mm^2^ substrate size), (**b**) microstructure of the multi-bead clad prepared for the section area at a distance of about 20 from the start of the bead.

In addition to the test results provided, multi-bead clads samples were also prepared for other service tests. They were deposited on rectangular 16Mo3 steel plates of the exact dimensions used for the single bead samples. The entire layer consisted of 36 beads with an offset of 2 mm (Figure 20a). The microstructure of the produced clad coating is shown in Figure 20b. The thickness of the layer was about 1.5 mm.

In the multi-pass clad, the successively deposited beads undergo multiple thermal cycles. Back-tempering occurs by the heat flow into areas previously hardened. The reheating of these areas makes the material to be back-tempered if the tempering temperature is reached. When the back-tempering occurs, a steel is characterized by a non-uniform microstructure and a lower hardness level. In the multi-pass cladding, it is a common practice to overlap each bead. This should avoid the soft boundary of base metal between the hardened ones. In this process, the back-tempering occurs at the beads’ intersection. Back-tempering can be controlled by using a proper amount of overlapping, scanning speed, and laser power. In the case of presented multi-pass clads, the overlapping was about 50%.

**Figure 21:**
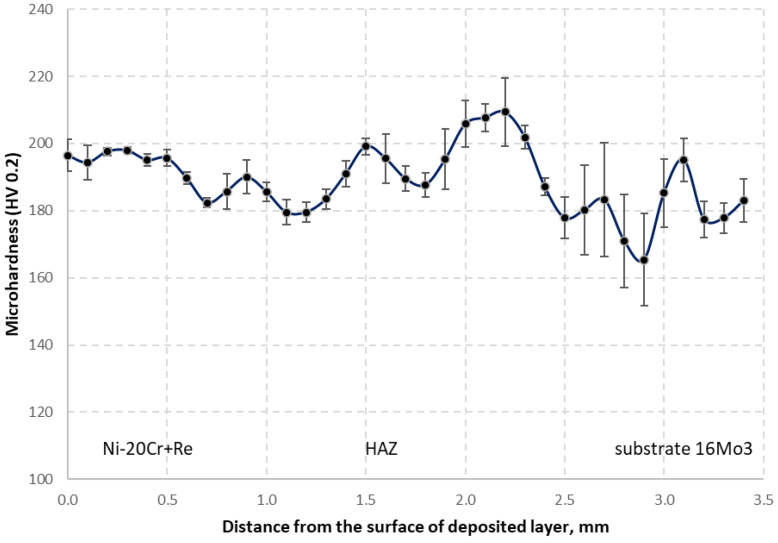
Microhardness distribution at the cross-sections of the deposited multi-bead Ni-20Cr+Re layer produced with 2400 W laser power.

In order to compare its impact on the deposited material, hardness measurements were taken in cross-section of the clad running perpendicular to the fusion line. Four series of measurements were carried out in the axis of each bead deposited with a 2 mm offset to each other. The results of the hardness distribution calculated based on these series of data are shown in Figure 21.

The hardness in the area of the clad is in the range from 175 to 205 HV, which is largely similar to the hardness measured for the single clads. A significant difference in the hardness distribution appears in the area of the heat-affected zone resulted from the back-tempering process. While in the case of a single bead, a sudden increase in hardness in the HAZ 1 and HAZ 2 zones to about 350–400 HV is visible, which is not observed for the multi-bead clad due to the back-tempering effect. Here, the hardness in the entire range of the heat-affected zone does not fluctuate much and remains in the range of 180–210 HV. In the base material and the transition zone to the HAZ, the measured hardness is within the limits of 165–195 HV. The significant decrease in hardness in the HAZ area for the multi-bead clad can be related to the multiple heat cycles and the process of back-tempering of the previously arranged beads. As an effect, a decrease in hardness in the HAZ area is visible.

## 4. Conclusions

In this work, the Ni-20Cr+Re composite powder with 1% (wt.) of Re was cladded onto the 16Mo3 steel substrates by laser with different values of power beam. The obtained clads were characterized by other shape parameters, varying dilution levels, and showed no evidence of defects. The macro and microstructure examination followed by EDS surface and linear element distribution in the deposit and HAZ, and microhardness analysis, were correlated with the power of the laser beam used.

The main conclusions that can be drawn based on the conducted research are as follows:

- Ni-20Cr+Re deposits obtained in the laser cladding process are characterized by the correct shape and do not show any visible defects. With a laser power of up to 1800 W, the clad does not melt into the substrate. Based on the macrostructure analysis, it was found that the most favorable clad shape occurs with a laser power of 2400 W. The microstructure of the clad showed no cracks, with about 16% dilution.
- X-ray diffraction tests did not show the presence of Rhenium in the layer due to the too low content of this element in the composite, and more detailed research should be undertaken to detect possible phases in the clad area.
- The microstructure observations of obtained deposits showed that we could distinguish in HAZ four sub-areas whose structure changed gradually from the fusion line towards the base material. The microstructure in the HAZ changes from martensitic, through martensitic–bainitic, to ferritic–bainitic microstructure. The length of these subzones depends on the cladding parameters. For the laser power of 2400 W, the first HAZ1 subzone was approximately 150 μm in length, while the others were around 200 μm each.
- The change of element concentration at the fusion line was gradual. That is accompanied by a significant expansion of the fusion line, promoting good metallurgical bonding between the deposit and the substrate material. The correct dilution coefficient should ensure adequate bonding and limit the amount of substrate material in the deposit. In produced Ni-20Cr+Re clads, good results were obtained for a deposit with approximately 16% dilution at a laser power of 2400 W.
- The EDS analysis of the element distribution did not show any sudden changes in the composition of the clad and the area of the fusion line. The structure of the clad was dendritic, evenly distributed. The content of Rhenium in the clad was determined at approximately 1% (wt.).
- The maximum hardness occurred in the HAZ, mainly in sub-areas HAZ 1 and HAZ 2, and reached approximately 350–400 HV at the laser power between 1500 and 2100 W. The decrease of the width of HAZ is observed with a reduction of the laser power.
- The analysis of the grain size distribution in the HAZ indicates that it may impact hardness changes in the zone between the fusion line and the base material. As we move away from the fusion line, grain fractions with increasingly larger diameters appear (lower G number), accompanied by a decrease in material hardness.
- In the case of a multi-bead clad layer, there is an apparent decrease in hardness in HAZ 1 and HAZ 2 compared to the values obtained for a single bead. This is a result of a back-tempering process initiated when next beads overlap the previously deposited ones. In a single-bead process, the steel transformation upon cooling results in metallurgical changes that increase the hardness in the HAZ subarea close to the fusion line.

## Figures and Tables

**Table 1:** Chemical composition of 16Mo3 steel.

Element	Fe	C	Si	Mn	P	S	Cr	Mo	Ni	N	Cu
wt.%	Bal.	0.12–0.2	0.35	0.4–0.9	0.025	0.01	0.3	0.25–0.35	0.3	0.012	0.3

**Table 2:** Chemical composition of Ni-20Cr alloy (Amdry 4535).

Element	Ni	Cr	Si	Fe	Mn	Others Max
wt.%	Bal.	19.5	0.75	0.25	0.25	0.4

**Table 3:** Geometrical parameters of laser cladded Ni-20Cr+Re beads measured on the cross-section of the samples.

Laser Power (W)	Height, h (µm)	Depth, d (µm)	Width, w (µm)	Dilution, D (%)
1500	913	0	2698	0.0
1800	1114	0	3154	0.0
2100	984	234	3559	8.5
2400	1005	320	3663	16.1
2700	1038	401	3881	20.8

## Data Availability

Data is contained within the article.
